# High prevalence of *Campylobacter jejuni* CC21 and CC257 clonal complexes in children with gastroenteritis in Tehran, Iran

**DOI:** 10.1186/s12879-021-05778-5

**Published:** 2021-01-23

**Authors:** Mahnaz Sarhangi, Bita Bakhshi, Shahin Najar Peeraeyeh

**Affiliations:** Department of Bacteriology, Faculty of Medical Sciences, Tarbiat Modares University, Jalal-Ale-Ahmad Ave, Tehran, 14117-13116 Iran

**Keywords:** *C. Jejuni*, LOS class, Capsular genotype, *dnaK*, MLST, Iran

## Abstract

**Background:**

*Campylobacter jejuni (C. jejuni*) is a leading cause of acute gastroenteritis in human worldwide. The aim of study was to assess the distribution of sialylated lipooligosaccharide (LOS) classes and capsular genotypes in *C. jejuni* isolated from Iranian children with gastroenteritis. Furthermore, the level of *dnaK* gene expression in *C. jejuni* strains with selected capsular genotypes and LOS classes was intended. Moreover, a comprehensive study of *C. jejuni* MLST-genotypes and inclusive comparison with peer sequences worldwide was intended.

**Methods:**

Twenty clinical *C. jejuni* strains were isolated from fecal specimens of 280 children aged 0–5 years, suspected of bacterial gastroenteritis, which admitted to 3 children hospitals from May to October, 2018. Distribution of sialylated LOS classes and specific capsular genotypes were investigated in *C. jejuni* of clinical origin. The expression of *dnaK* in *C. jejuni* strains was measured by Real-Time-PCR. MLST-genotyping was performed to investigate the clonal relationship of clinical *C. jejuni* strains and comparison with inclusive sequences worldwide.

**Results:**

*C. jejuni* HS23/36c was the predominant genotype (45%), followed by HS2 (20%), and HS19 and HS4 (each 10%). A total of 80% of isolates were assigned to LOS class B and C. Higher expression level of *dnaK* gene was detected in strains with HS23/36c, HS2 and HS4 capsular genotypes and sialylated LOS classes B or C. MLST analysis showed that isolates were highly diverse and represented 6 different sequence types (STs) and 3 clonal complexes (CCs). CC21 and CC257 were the most dominant CCs (75%) among our *C. jejuni* strains. No new ST and no common ST with our neighbor countries was detected.

**Conclusions:**

The *C. jejuni* isolates with LOS class B or C, and capsular genotypes of HS23/36, HS2, HS4 and HS19 were dominant in population under study. The CC21 and CC257 were the largest CCs among our isolates. In overall picture, CC21 and CC353 complexes were the most frequently and widely distributed clonal complexes worldwide, although members of CC353 were not detected in our isolates. This provides a universal picture of movement of dominant Campylobacter strains worldwide.

## Introduction

*Campylobacter jejuni (C. jejuni*) is a leading cause of bacterial foodborne poisoning and acute gastroenteritis in human worldwide [1]. This bacterium often causes a moderate to severe watery regularly self-limiting gastroenteritis and post-infectious immune disorders such as Guillain-Barre syndrome (GBS) [2].

*C. jejuni* produces Capsular Polysaccharide (CPS). The CPS gene cluster is located in a hypervariable region in the *C. jejuni* genome [3]. Major serodeterminant of the CPS is in classical Penner or Heat-Stable (HS) serotyping Scheme. In the classical Penner serotyping scheme, which is based on the passive slide hemagglutination assay, *C. jejuni* strains are divided into 47 serotypes, of which, due to similarity in the CPS structure, 35 serotypes have been refined, which are serotype cross-reactive pairs or complexes [4, 5]. *C. jejuni* Penner serotypes associated with GBS often belong to HS1, HS4c, HS19, HS23/36c and HS41, furthermore, the most common serotypes among sporadic cases are reported in the HS4c, HS2 and HS1 [4]. Recently, CPS genotyping was used as a more effective method, since it is not affected by variations in capsular gene expression which is observed in serotyping [2]. Moreover, CPS genotyping is a fast, accessible and reliable method to determine CPS types in *C. jejuni* [2, 3].

The cluster of genes involved in *C. jejuni* lipooligosaccharide (LOS) biosynthesis, is one of the most variable regions of *C. jejuni* genome [6] due to mutations within or recombination between LOS biosynthesis gene/gene regions [7].

Among 19 different LOS locus classes from A to S, 3 classes (A, B and C) play a key role in the biosynthesis of the sialic acid and are often isolated from the stools of patients with GBS [2, 6, 8].

Post-infection diseases like GBS with *C. jejuni* have been proved to be associated with antibodies of human gangliosides. The induction of these autoantibodies is associated with molecular mimicry between human gangliosides and bacterial epitopes present at the surface of the LOS [9]. It is worth noting that the antibody responses to gastroenteritis is different to GBS triggered by *C. jejuni* [10]. In addition to antiganglioside antibodies, Heat Shock Proteins (HSP) family can mediate in the autoimmune diseases. They belong to a highly protected family that is present in normal physiological conditions in prokaryotic and eukaryotic cells. These proteins are etiologic factors in many autoimmune diseases in such a way that their overexpression leads to environmental stress induction [9, 11].

*C. jejuni* carry several of HSPs, including *groELS*, *dnaJ*, *dnaK* and *lon* [12] among which DnaK proteins (70 kDa) has a high sequence homology with HSP70 of human peripheral neurons [9]. A high titer of anti-HSP antibody (HSP27, HSP60 and HSP70) can be found in CSF (Cerebrospinal fluid) of patients with GBS [9].

To date, no study has been reported on the distribution of CPS genotypes, LOS locus classes and Multilocus sequence typing (MLST) of *C. jejuni* in Iran. The aim of our study was to assess, for the first time, the distribution of sialylated LOS classes and capsular genotypes among clinical *C. jejuni* strains isolated from Iranian children with gastroenteritis. Moreover, the correlation of DnaK protein expression level in *C. jejuni* strains with selected capsular genotypes and LOS classes was intended. Furthermore, a comprehensive comparison of *C. jejuni* MLST genotypes with peer sequences reported worldwide was envisioned.

## Materials and methods

### Phenotypic and genotypic identification of *C. jejuni* strains from fecal samples

Based on Cochran formula for calculating of sample size, a total of 3000 gastroenteritis cases were examined for suspected cases of sporadic campylobacteriosis. Gastroenteritis was characterized as abdominal pain with ≥3 episodes per day. Children with underlying gastrointestinal disease, physiologic diarrhea or history of antibiotic intake were excluded, which were defined by physicians in hospitals. Suspected cases of bacterial gastroenteritis were subjected to specimen collection, among which 280 cases accompanied WBC (white blood cell) and RBC (red blood cell) shedding in the majority of cases and were considered as suspected cases of campylobacteriosis.

Fecal specimens were collected from children with gastroenteritis, aged 0–5 years, referred to 3 Children’s Medical Center and Hospitals at Tehran, Iran, from May to October 2018. Information on age, clinical symptoms, history of non-pasteurized dairy products consumption, animal contact as well as laboratory results was recorded. Then specimens were transferred from laboratory of hospital to the laboratory of Tarbiat Modares University using Carry-Blair Transport Media (Micro Media-Hungary) and immediately streaked on Brucella agar and modified charcoal-cefoperazone-deoxycholate agar (mCCDA) (Merck-Germany). Plates were incubated at 42 °C for 48 h under microaerophilic condition using Gas Pack C (Merck-Germany). Gram staining, spiral morphology, catalase and oxidase production, nitrate reduction and indoxyl acetate hydrolysis test were used to confirm *C. jejuni* colonies. Also, hippurate hydrolysis test was used for phenotypic distinguishing of *C. jejuni* from *C. coli* from enteritis patients. Eventually, twenty *C. jejuni* and three *C. coli* strains were confirmed by Duplex PCR [13] and in the following, twenty *C. jejuni* was studied.

Twenty-three *Campylobacter* isolates were identified from 280 stool samples among which 20 and 3 isolates were *C. jejuni* and *C. coli*, respectively. All confirmed *C. jejuni* strains (*n* = 20) were designated and used for further analyses.

### Identification of capsular genotypes and LOS locus classes

The capsular genotypes of *C. jejuni* strains were identified using specific primers for most commonly found genotypes HS1, HS2, HS4c, HS19, HS23/36c and HS41 (Table 1) [4, 5].

**Table 1:** The primer sequences included in the *C. jejuni* capsule typing scheme

Primer	Forward sequence	Reverse sequence	Product size (bp)	Reference
HS1	GCAAGAGAAACATCTCGCCTA	TTGGCGGTAAGTTTTTGAAGA	610	[5]
HS2	CATCCTAGCACAACTCACTTCA	CAGCATTGGAGGATTTACAATATAT	62	[5]
HS4A	CCTAACATATCATACACTACGGT	TATATTTGGTTAGGGATCCA	370	[5]
HS19	GGCAACAAACAAACATATTCAGA	CGAGGATGAAAATGCCTCAA	450	[5]
HS23/36	GCTTTATATCTATCCAGTCCATTATCA	GCTTGGGAGATGAATTTACCTTTA	161	[5]
HS41	TGCAATCTCTAAAGCCCAAG	CTTACATATGCTGGTAGAGATGATATG	279	[5]

From 19 different LOS locus classes from A to S, 3 classes (A, B and C) play a key role in the biosynthesis of the sialic acid and are often found in isolates from the stools of patients with GBS [2, 6, 8]. Therefore, sets of primers specific for class A, B, and C classes were used for characterization of LOS identities (Table 2) [6].

**Table 2:** Primer used for identification of classes A, B and C

ORF	Forward sequence	Reverse sequence	Product size (bp)	Reference
Orf7ab	ACTACACTTTAAAACATTTAATCC AAAATCA	CCATAAGCCTCACTAGAAGGTATGAGTATA	580	[6]
Orf 6ab1	CAAGGGCAATAGAAAGCTGTATCA	ACAAGCACTTCATTCTTAGTATTACAAAT	631	[6]
Orf6ab2	TCATCTTGCCAACTTATAATGTGGA	TCTAGCGATATTAAACCAACAGCCT	517	[6]
Orf5bII	CTGTGATGATGGGAGTGAAGAGC	GGTAATCGTTTCGGCGGTATT	539	[6]
Orf6c	GTAGTAGATGATTGTGGTAATGATAAA	ATAGAATTGCTATTTACATGCTGG	554	[6]
Orf7c	TTGAAGATAGATATTTTGTGGGTAAA	CTTTAAGTAGTGTTTTATGTCACTTGG	746	[6]

Genomic DNA was extracted using a genomic DNA extraction kit (GeNetBio, Korea), according to the manufacturer instructions. PCRs was carried out within a thermal cycler (Eppendorf, Germany) in a final volume of 25 μL containing 1–10 ng DNA template, 2.5 μL 10X PCR buffer, 1 unit of Taq DNA polymerase, 2.0 mM MgCl_2_, 0.2 μM of each primer, 0.3 mM each dNTP and sterile deionized water. Amplification conditions was as follow: 95 °C for 5 min, followed by 30 amplification cycles including denaturation at 94 °C for 1 min, annealing at 52 °C for 1 min and extension at 72 °C for 1 min. The reaction was ended with a final extension at 72 °C for 5 min and followed by electrophoresis of amplicons on 1% agarose gel.

### Real-time PCR for *dnaK* gene expression in clinical *C. jejuni* isolates

Microbial DnaK is a bacterial conserved chaperone protein which has a sequence homology with human peripheral nerve HSP70 and its high level expression is supposed to be related to GBS promotion [14, 15].

In order to assess the efficiency of real-time PCR amplification, five serial 1:10 dilutions of cDNA was used as template for qRT-PCR reaction of the *dnaK and* 16srRNA genes. The CTs values and the concentrations of the template were used to plot the standard curve and calculate the primer efficiency.

RNA extraction was performed on 20 *C. jejuni* strains, which were previously checked for the presence of capsular types and LOS locus classes using a Favoren Biotec Corp kit (Taiwan). Subsequently, the RNA molecules were treated using the DNase I kit (TaKaRa). A cDNA synthesis kit (Yekta Tajhiz Azma-Iran) was used to generate a single-strand cDNA. The cDNAs were kept at − 20 °C. Quantitative Real Time-PCR was performed using SYBR Green (RealQ Plus Master Mix Green-Denmark) in Qiagen-Rotor-Gene Q with HRM. 16S rRNA gene was used as the internal control. One of the isolates that neither had the selected capsular serotypes nor the LOS locus classes was considered as a reference gene. The PCR reaction mixture consisted of 100 ng to1 mg of cDNA (for *dnaK* and 16S rRNA genes), 1 mM of each primer (Table 3) [16] and 12.5 mL of SYBR Green I Master Mix. Cycling conditions included an initial denaturing step of 10 min at 95 °C followed by 40 cycles of 15 s at 95 °C and 1 min at 60 °C. 2^-∆∆*Ct*^ is a relative quantification method for analyze the relative changes in gene expression from real-time quantitative PCR [16]. SPSS software version 20 was used for the analysis of data.

**Table 3:** Specific primers used for Real-Time PCR

Primer	Forward sequence	Reverse sequence	Product size (bp)	Reference
*dnaK*	AAACGCCAAGCGGTAACTAA	TTCTTTAGCCGCGTCTTCAT	90	[16]
16SrRNA	AAGGGCCATGATGACTTGACG	AGCGCAACCCACGTATTTAG	107	[16]

### Multilocus sequence typing (MLST)

The MLST method was performed according to Dingle, et al. [17]. Each 25 μL amplification reaction mixture comprised 1–10 ng DNA template, 1X PCR buffer, 1.25 unit of Taq DNA polymerase, 1.5 mM MgCl_2_, 1 μM of each primer (Table 4), 0.8 mM each dNTP and sterile deionized water.

**Table 4:** Primers used for *Campylobacter jejuni* MLST

Primer	Forward	Reverse	Product size (bp)	Reference
*asp*	CCAACTGCAAGATGCTGTACC	TTAATTTGCGGTAATACCATC	625	[17]
*gln*	CATGCAATCAATGAAGAAAC	TTCCATAAGCTCATATGAAC	722	[17]
*glt*	GTGGCTATCCTATAGAGTGGC	CCAAAGCGCACCAATACCTG	575	[17]
*gly*	AGCTAATCAAGGTGTTTATGCGG	AGGTGATTATCCGTTCCATCGC	648	[17]
*pgm*	GGTTTTAGATGTGGCTCATG	TCCAGAATAGCGAAATAAGG	700	[17]
*tkt*	GCTTAGCAGATATTTTAAGTG	ACTTCTTCACCCAAAGGTGCG	691	[17]
*unc*	TGTTGCAATTGGTCAAAAGC	TGCCTCATCTAAATCACTAGC	631	[17]

The PCR conditions were as follows: denaturation at 94 °C for 2 min; annealing at 50 °C for 1 min, extension at 72 °C for 1 min for 35 cycles. DNA Sequences of each housekeeping gene were submitted to *C. jejuni* MLST database (http://pubmlst.org/campylobacter) and the related allelic numbers, Sequence Types (ST) and Clonal Complexes (CC) were identified [18]. The Accession Numbers of DNA Sequences of 20 *C. jejuni* isolates were deposited in GenBank (https://pubmlst.org/campylobacter). Dendrogram was plotted using Interactive Tree Of Life (iTOL) v4 [19].

### Geographic distribution of STs and CCs

A circular dendrogram was plotted for comparison of the peer sequences reported worldwide. A total of 72,392 isolates were downloaded from PubMLST website and analyzed [18]. Based on the inclusion and exclusion criteria, a total of 304 isolates were included in dendrogram. The inclusion criteria were: *C. jejuni* strains from the five recent years (2014–2018), *C. jejuni* strains from human stool, *C. jejuni* strains from the sporadic cases and gastroenteritis. The inclusion criteria were based on parameters that well warrant comparisons with our strains. Excluding criteria were unspecified ST or CC as well as repeated ST samples of each country.

The recorded MLST data in PubMLST database were also used to compare STs and CCs between our isolates with those from our neighbor countries. Among Iran neighboring countries only Turkey and Pakistan had recorded data about *C. jejuni* in PubMLST. Eleven isolates from human stool samples has been reported form these countries which were included for the final analysis. Dendrograms was plotted using Interactive Tree Of Life (iTOL) v4 [19]. Various sequence types were obtained from and plotted using PubMLST database tools [18].

### Statistical analysis

To assess the presence of sialylated LOS classes A, B and C and CPS types in 20 *C. jejuni* strains isolated from children with gastroenteritis, we used the Cochran’s Q test. Data were analyzed with the Statistical Package for Social Sciences (Version 25.0, SPSS Inc., Chicago, IL, USA); *p* < 0.05 was considered statistically significant.

## Results

Twenty-three *Campylobacter* isolates were identified from 280 stool samples of children with gastroenteritis. Among the 23 isolates, 20 and 3 isolates were *C. jejuni* and *C. coli*, respectively, based on hippurate hydrolysis test and duplex PCR assay of *cadF* gene. Analysis of the duplex PCR assay of *cadF* gene showed that 737 and 461 bp amplicons were corresponding to *C. jejuni* and *C. coli*, respectively. All confirmed *C. jejuni* strains (*n* = 20) were designated and used for further analyses.

### CPS genotype and LOS locus class diversity

From 20 *C. jejuni* isolates, 17 (85%) expressed one of the selected CPS genotypes under study. CPS types HS23/36c were found in 9 isolates (45%) and appeared as dominant CPS among 20 *C. jejuni* strains (sig = 0.001; Cochran’s Q test). HS2, HS19, and HS4 were detected in 4 (20%), 2 (10%) and 2 (10%) isolates, respectively. No *C. jejuni* isolate belonged to HS1 or HS41 genotypes.

Of 20 strains, 16 (80%) expressed sialylated LOS locus class B or C. The LOS class B with 11 isolates (55%) was dominant sialylatd LOS class (sig = 0.003; Cochran’s Q test). No isolate with LOS class A was found among our isolates.

### Relationship between CPS type and LOS locus class

**Fig. 1:**
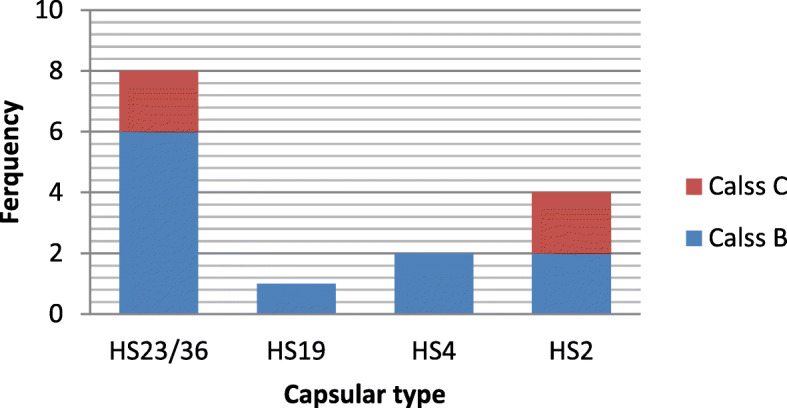
Relationship between presence of CPS type genes and LOS locus class genes

Among *C. jejuni* strains with LOS class B, the distribution of CPS types was as follows: HS23/36c (*n* = 6, 54/54%), HS2 (*n* = 2, 18/18%), HS4 (*n* = 2, 18/18%) and HS19 (*n* = 1, 9/9%). Also, the distribution of CPS genotypes among *C. jejuni* strains with LOS class C was as HS23/36c (*n* = 2, 40%) and HS2 (*n* = 2, 40%) (Fig. 1).

### Comparison of *dnaK* gene expression in clinical *C. jejuni* strains

**Fig. 2:**
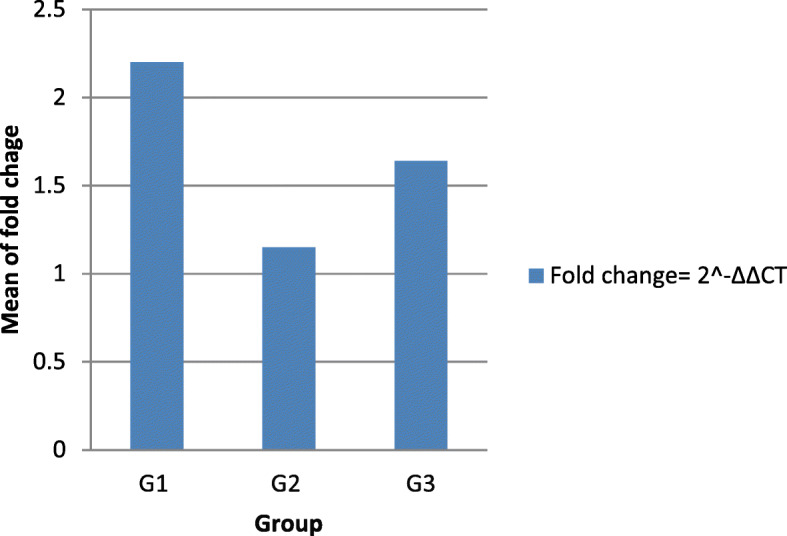
Level of *dnaK* gene expression in clinical *C. jejuni* isolatesof different groups

The primer efficiency was calculated as 97.51% and 103.54 for *dnaK* and 16srRNA genes, respectively. The *dnaK* gene expression was determined by Quantitative Real-Time PCR and according to the 2^-ΔΔCT^ method. Among isolates that showed one of the LOS classes of A-C or one of six selected capsular genotypes, 18 isolates were classified into three groups including group 1: with identified CPS genotype and sialylated LOS class (B or C) (*n* = 15), group 2: with identified CPS genotype and without sialylated LOS class (*n* = 2), group 3: without CPS genotype but with sialylated LOS class (*n* = 1), and one of the isolates that neither had the selected capsular serotypes nor the LOS locus classes was considered as a reference strain. Due to insufficiency of data, differential expression analysis could not be performed; thus, descriptive statistics was used instead. As a result, *dnaK* expression level in group 1 was greater than other groups (Table 5). The *dnaK* expression level was much higher in clinical *C. jejuni* isolates with one of the CPS genotypes and the LOS classes relevant to GBS patients. Based on the 2^-ΔΔCT^ method, the graph of the fold change of *dnaK* gene expression was plotted (Fig. 2).

**Table 5:** Relationship between the level of *dnaK* gene expression in clinical *C. jejuni* isolates

Group	Isolate ID	Capsular genotype	LOS class	Fold Change: 2^-∆∆CT^
G1	79,434	HS2	B	2.04 ± 0.72
G1	79,425	HS23/36	B	1.54 ± 0.44
G1	79,426	HS23/36	B	1.34 ± 0.3
G1	79,438	HS23/36	B	5.43 ± 1.7
G1	79,420	HS2	C	2.66 ± 0.99
G1	79,437	HS23/36	B	3.0 ± 1.1
G1	79,427	HS4	B	2.16 ± 0.78
G1	79,435	HS2	B	2 ± 0.7
G1	79,439	HS23/36	B	1.5 ± 0.41
G1	79,444	HS23/36	C	1.6 ± 0.49
G1	79,443	HS4	B	1.70 ± 0.53
G1	79,253	HS23/36	C	1.02 ± 0.02
G1	79,429	HS19	B	1.34 ± 0.29
G1	79,440	HS23/36	B	3.68 ± 1.3
G1	79,421	HS2	C	2.65 ± 0.99
				Total: Mean of means: 2.2 ± 0.71
G2	79,441	HS23/36	–	1.1 ± 0.12
G2	79,428	HS19	–	1.21 ± 0.19
				Total: Mean of means: 1.15 ± 0.15
G3	79,442	–	C	1.64 ± 0.45
Reference	79,436	–	–	1 ± 0

G1: Isolates with capsular genotype and LOS class

G2: Isolates with capsular genotype and without LOS class

G3: Isolates without capsular genotype and with LOS class

### CC and ST variation of MLST analysis

Based on the MLST analysis of 20 isolates, 6 sequence types and 3 clonal complexes were detected in Iran. Five and 7 isolates were identified as ST-257 and ST-50, respectively. Each of the ST-19 and ST-5326 were detected in three isolates, while ST-1096 and ST-1113, each were only detected in one isolate.

Out of the 3 identified clonal complexes, ST-21 complex dominated in 10 isolates (50%).

Both ST-257 complex and ST-828 complex were found in 5 (25%) and 2 (10%) isolates, respectively.

**Fig. 3:**
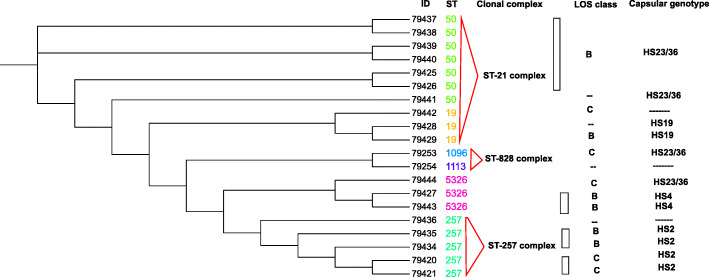
Dendrogram demonstrating the phylogenetic relationship between the 20 *C. jejuni *isolates from patients with gastroenteritis in Iran 2018. *Dendrogran plotted by Interactive Tree of Life (iTOL) v4 free access mode [19]

**Fig. 4:**
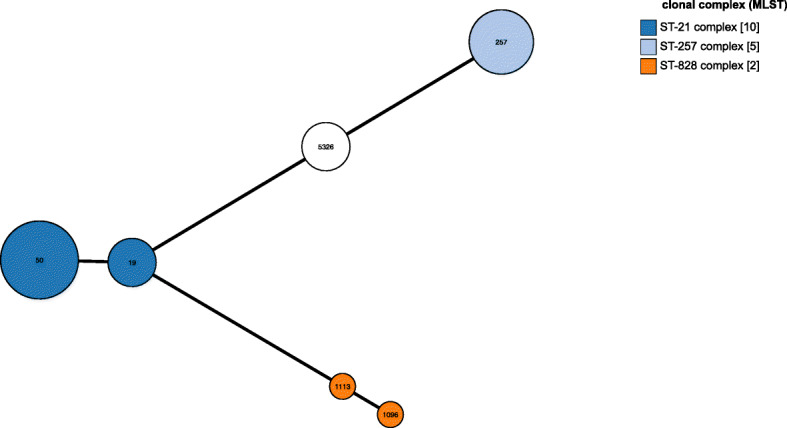
Minimum spanning tree for categorical data (based on clonal complexes) The tree was created using GrapeTree [20]. Each clonal complex is represented by a circle, numbers in each circle related to STs. the number of isolates is shown in brackets

Distribution of the sequence types and the genetic link between *C. jejuni* strains isolated from patients with gastroenteritis has been shown in the dendrogram (Fig. 3). The relationship among 20 isolates based on clonal complexes is reflected in minimum spanning tree diagram (Fig. 4) [20].

### Linkage between CPS genotypes and LOS class with MLST CC

**Fig. 5:**
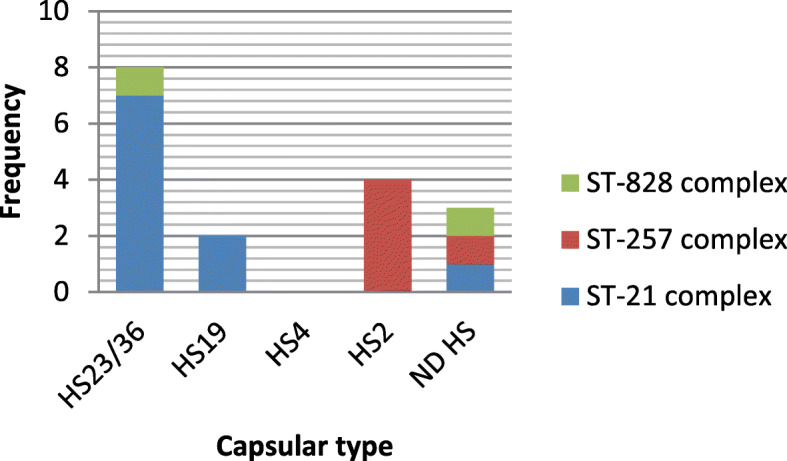
Linkage between CPS genotypes and MLST CC

**Fig. 6:**
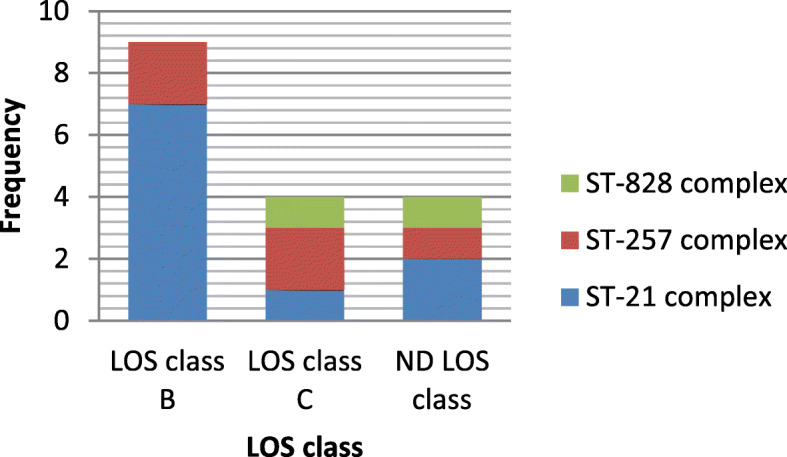
Linkage between LOS class and MLST CC

Totally, 3 CCs (CC828, CC257 and CC21) were identified. The isolates with HS23/36c and HS19 genotypes were found in CC21 (Fig. 5). Majority of isolates in CC21 had LOS class B or LOS class C. In CC257, 4 isolates belonged to HS2 serotype. In this CC, LOS class C and B were observed. Two isolates were assigned to CC828 (Fig. 6).

### Geographic distribution of STs and CCs

**Fig. 7:**
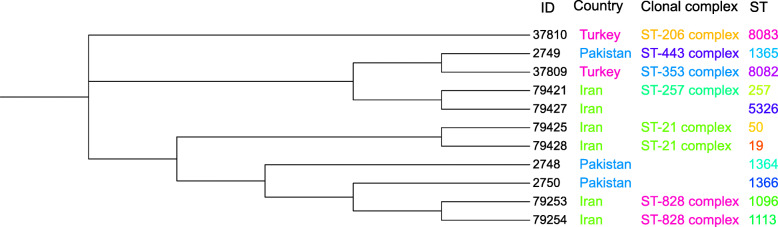
Phylogenetic analysis of 11 *C. jejuni* strains for Iran, Turkey and Pakistan. *Dendrogran plotted by Interactive Tree of Life (iTOL) v4 free access mode [19]

**Fig. 8:**
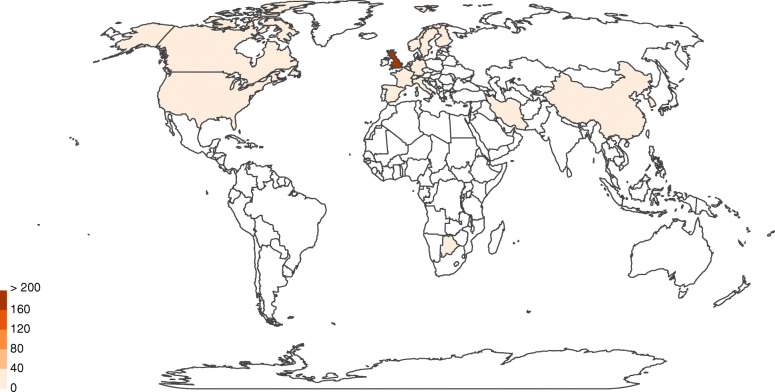
Geographic distribution of ST-19 for 1,324 *Campylobacter* isolates (*C. jejuni* (99.8%) and *Campylobacter spp* (0.2%) in the world, 1982-2018. https://pubmlst.org/campylobacter [18]

**Fig. 9:**
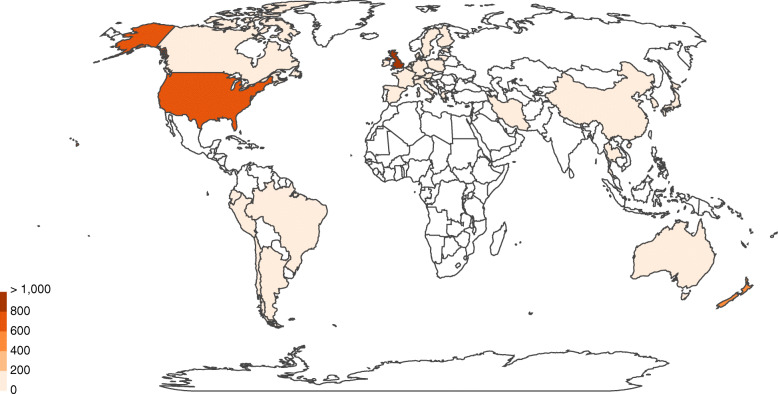
Geographic distribution of ST-50 for 3,868 *Campylobacter* isolates (*C. jejuni* (99.6%) and *Campylobacter spp* (0.4%) in the world, 1980-2018. https://pubmlst.org/campylobacter [18]

**Fig. 10:**
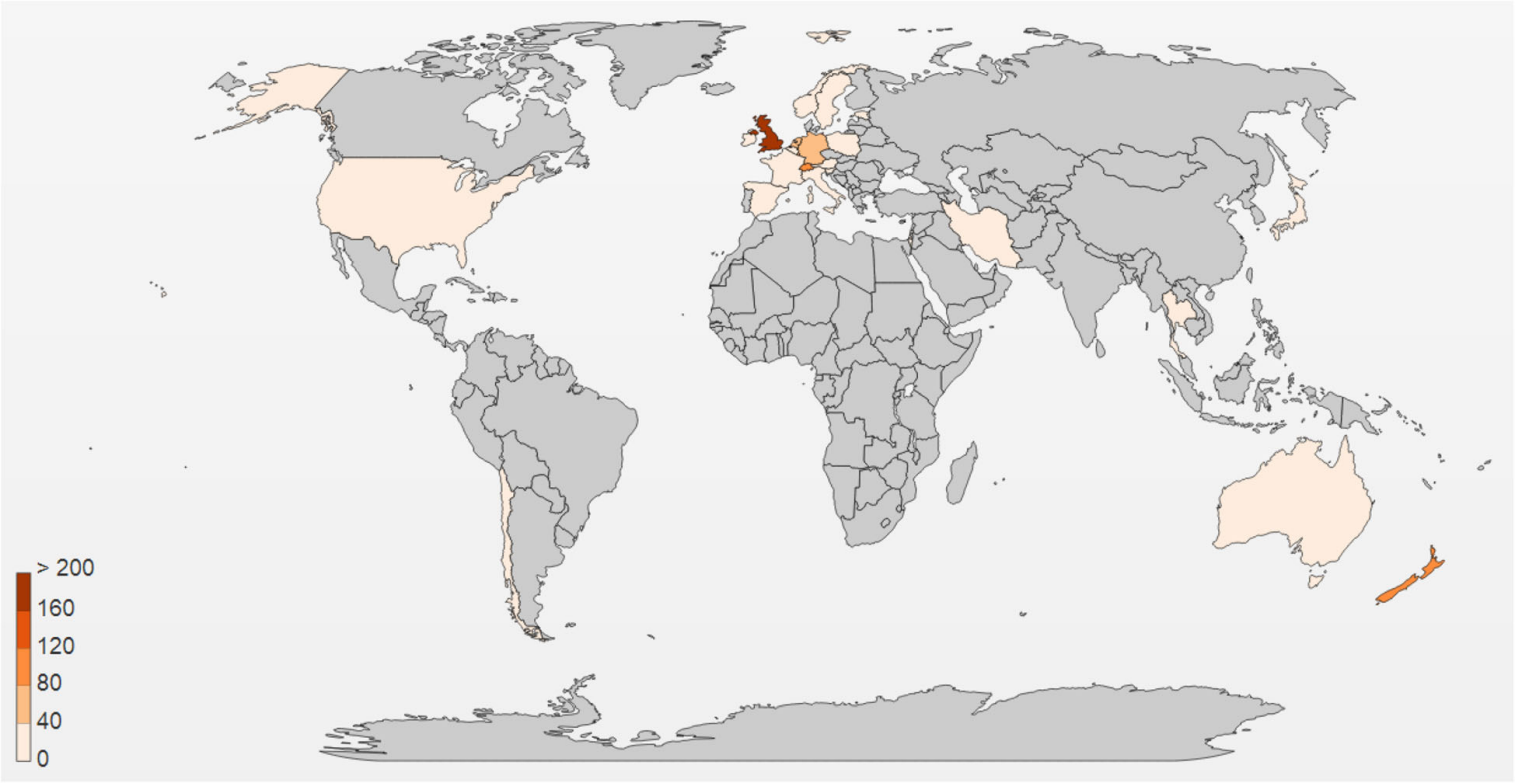
Geographic distribution of ST-257 for 2,689 *Campylobacter* isolates (*C. jejuni* (100.0%) in the world, 1990-2018. https://pubmlst.org/campylobacter [18]

A phylogenetic analysis revealed that there was no common STs or CCs between Iran and its neighbor countries, Turkey and Pakistan (Fig. 7). This may be due to scarcity of published data from these countries. No data is available from other neighboring countries of Iran. World map related to *C. jejuni* strains from 1980 to 2018 shows the distribution of ST19 (CC21), ST50 (CC21), and ST257 (CC257) among different continents (Figs. 8, 9 and 10). The frequency of CC21 strains was associated to sporadic cases of human gastroenteritis which was recorded from America (USA and Canada), Europe (UK, The Netherlands and Germany), Asia (Iran, this study) and Australia. Inclusive comparison of CC257 (including ST-257) strains in the same time period showed recorded strains from Asia (Iran, this study), Europe (UK and Spain), America (Chile) and Africa (South Africa).

**Fig. 11:**
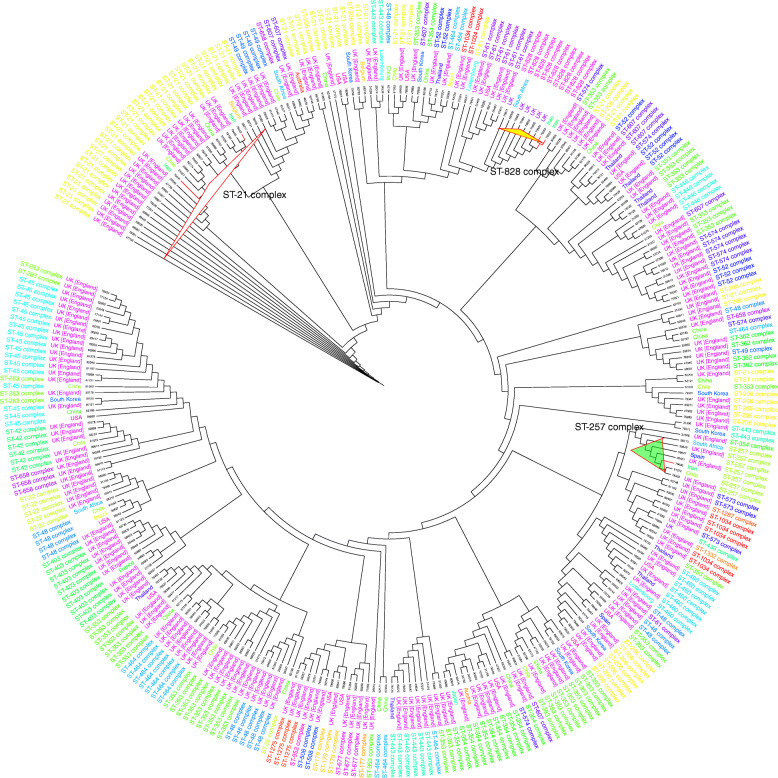
Phylogenetic analysis of 304 *C. jejuni *isolates worldwide from PubMLST database. *Dendrogran plotted by Interactive Tree Of Life (iTOL) v4 free access mode [19]. White triangle: Countries with similar CC21, yellow triangle: Countries with CC828 and green triangle: Countries with similar CC257 with Iran

In a dendrogram constructed by inclusive comparison of MLST results during 2014–2018 a similar picture was depicted for distribution of CC21 and CC257 clonal complexes. Moreover, neighbor-joining results indicated that CC21 and CC353 complexes are the most divers, most frequent and most widely distributed clonal complexes around the world, respectively; although, CC353 was not detected in the current study (Fig. 11) (Table 6).

**Table 6:** Top 10 Clonal complexes based on frequency and diversity extracted from circle dendrogram

Rank	Clonal Complex	Diversity	Rank	Clonal Complex	Frequency
1	ST-353 Complex	12	1	ST-21 Complex	51
2	ST-21 Complex	10	2	ST-353 Complex	42
3	ST-206 Complex	7	3	ST-206 Complex	17
4	ST-48 Complex	6	4	ST-45 Complex	17
5	ST-464 Complex	5	5	ST-48 Complex	14
6	ST-52 Complex	5	6	ST-354 Complex	13
7	ST-574 Complex	5	7	ST-464 Complex	13
8	ST-607 Complex	5	8	ST-403 Complex	10
9	ST-354 Complex	4	9	ST-443 Complex	10
10	ST-1034 Complex	3	10	ST-52 Complex	9

## Discussion

*Campylobacter spp*. is considered to be the most common bacterial cause of human gastroenteritis in the world [21]. We aimed to investigate CPS types, sialylated LOS classes and MLST types of virulent *C. jejuni* strains isolated from children with gastroenteritis aged 0–5 years in Tehran, Iran.

Collection time of our isolates was between May and October. Epidemiological reports from other countries [22, 23] suggest that human campylobacteriosis tends to increase during this period of year.

Epidemiological investigations have shown that a *C. jejuni* infection precedes GBS in 20 to 50% of cases in Europe, North and South America, Japan, and Australia [23]. Sialylated LOS loci of A, B and C classes as well as HS types of HS2, HS4, HS23/36c and HS19 are accused to be associated with GBS patients [2, 23, 24]. The prevalence of LOS class B was (11/20; 55%), followed by class C (5/20; 25%), while class A was not detected. Serichantalergs et al., also reported the sialylated LOS classes A, B and C in children with gastroenteritis which is consistent with our results [25].

Dominant CPS genotype among our isolates was HS23/36c (9/20) followed by HS2 (4/20). In an overall picture depicted by Pike et al., these two genotypes, after HS4, comprise the most prevalent CPS types worldwide [26]. Consistent with our findings, Sainato et al. identified HS2 and HS4 as two of eight most prevalent CPS types in pediatric population with gastroenteritis in Egypt [27]. Two CPS types of HS1 and HS41 were not detected among our 20 *C. jejuni* isolates. But, according to the report of Pike et al., HS1 was one of the dominant CPS types in the overall picture [26]. This may be due to the limited period and small number of our isolates compared with isolates from various populations and in a longer time period.

A wide range of CPS types including HS1/44, HS2, HS4, HS19 and HS23/36c are usually identified in *C. jejuni* strains isolated from GBS patients [2, 24]. In our gastroenteritis-related samples 85% of strains expressed one of HS23/36c, HS2, HS4 and HS19, which signifies the high probability of GBS progress in this group of patients.

In Iran, studies reported non-polio acute flaccid paralysis (AFP) incidence rates ranging from 0.3 to 6.5 per 100,000 [28]. The annual incidence of GBS is 0.6–4 cases per 100,000 populations that is a most common cause of nono-polio worldwide. Also in Iran, GBS is the most common cause of paralysis among AFP patients [29–31]. Almost 25–40% of GBS patients worldwide suffer from *C. jejuni* infection 1–3 weeks prior to the illness [32]. We demonstrated that *C. jejuni* isolates had the predominant LOS class B and C, and capsular genotypes of HS23/36, HS2, HS4 and HS19, all of which are accused to be associated with the progress of gastroenteritis toward GBS. Therefore, attention to incidence of *C. jejuni*, together with proper identification and treatment of children with campylobacteriosis is necessary for prevention of subsequent GBS.

Our findings showed that the *dnaK* expression mean in strains with specified capsular genotype and sialylated LOS was greater than that of others (with either capsular genotype or LOS class). Furthermore, our finding revealed that the expression of *dnaK* was higher when sialylated LOS and particular capsular genotypes simultaneously are present in a strain. Moreover, it was shown by HU et al., that *dnaK* gene expression is upregulated in conditions simulating in-vivo which means it may be induced in infected human host. Considering the crucial role of *dnaK* in antigenic mimicry and GBS, it can be concluded that individuals infected with *C. jejuni* strains having sialylated LOS classes and the selected capsular serotypes as well as a high expression profile of *dnaK* may be more likely to develop GBS. Furthermore, *dnaK* gene can be mentioned as an antigen candidate in preventive studies or as a diagnostic marker [10].

Genetic variations in 20 *C. jejuni* strains from enteritis patients was also identified by MLST. A total of 6 STs were observed and 17/20 (85%) belonged to 3 clonal complexes (CCs), while 3 isolates belonged to STs unassigned to a CC.

The majority of *C. jejuni* strains were assigned to CC21; this finding supports previous observations which shows CC21 is the most prevalent CC worldwide [33]. Within CC21, ST-50 was the dominant ST in our clinical samples, although not all of previous studies reported ST-50 as the dominant sequence type [34, 35].

Meanwhile, consistent with our finding, ST-50 and ST-19 (CC21) and ST-257 were mostly related to human campylobacteriosis cases [36–38]. However, isolates from other sources (fresh whole retail chicken, raw milk and environmental water) also presented CC21 as dominant CCs [39, 40].

The correlation between certain MLST clonal complexes and LOS classes and HS types were investigated in present study. The majority of *C. jejuni* isolates in ST-21 (7/10; 70%) expressed LOS class B, while both LOS class B and C occurred in ST-257 complex in an equal percentage (2/5; 40%). Habib et al., demonstrated that ST-21 complex strongly correlated with LOS class C [23] but this combination was rare among our isolates.

MLST analysis demonstrated that ST-21 and ST-257 complexes were dominants in our enteritis *C. jejuni* strains. Overall of different studies, the ST-22 complex was significantly overrepresented in the GBS isolates and ST-21 complex in enteritis isolates [2, 24, 35]. No new sequence type was detected in present study. Moreover, among neighbor countries, only a few data from Pakistan and Turkey was available and phylogenetic analysis revealed no common ST or CC with Iran.

Inclusive comparison of MLST results demonstrated that ST-50 (CC21) was widely distributed in different countries including UK, USA, Canada, some European countries, Australia and China, while ST-19 (CC21) and ST-257 (CC257) was less ubiquitously spread and absent from Australia and China/Canada, respectively.

Moreover, neighbor-joining results indicated that CC21 and CC353 are the most diverse, most frequent and most widely distributed clonal complex around the world; although, CC353 was not detected in current study. The most diverse CCs are related to more prevalent sequence types. This proposes that probably their diversity is a mirror of their replication frequency and circulation which affects their gene content and efficiency. This shows the movement of *C. jejuni* strains beyond the boundaries. The occurrence of identical clonal complexes with different capsular types and LOS classes is consistent with genetic variation in circulating identical genotypes and their evolution toward different pathotypes probably through acquisition of different genetic elements including LOS and CPS gene clusters.

## Conclusions

To our knowledge, this is the first report identifying CPS types and sialylated LOS classes, as well as MLST genotyping of *C. jejuni* strains related to gastroenteritis of children in Tehran, Iran. We demonstrated that i) *C. jejuni* isolates had the predominant LOS class B and C, and capsular genotypes of HS23/36, HS2, HS4 and HS19, all of which are accused to be associated with the progress of disease toward GBS; ii) Higher expression level of *dnaK* gene was detected in strains with HS23/36c, HS2 and HS4 capsular genotypes and sialylated LOS classes B or C, therefore its expression can be used as an indicator for probable GBS progress in infected patients; iii) Isolates were highly genetically diverse and distributed in 6 STs. CC21 and CC257 were predominant in our isolates. Comprehensive comparison of MLST results demonstrated that CC21 is the largest clonal complex of *C. jejuni* strains worldwide and provides a universal picture of movement of dominant Campylobacter strains.

## Data Availability

The datasets of the current study are available within article or can be obtained from corresponding upon request. The Accession Numbers of DNA sequences of 20 *C. jejuni* isolates were deposited in GenBank at https://pubmlst.org/bigsdb?db=pubmlst_campylobacter_isolates&page=query. The values are as follows: 79420, 79421, 79425, 79426, 79427, 79428, 79429, 79434, 79435, 79436, 79437, 79438, 79439, 79440, 79441, 79442, 79443, 79444, 79253 and 79254.

## Abbreviations

*C. jejuni*: *Campylobacter jejuni*
CPS: Capsular Polysaccharide
HS: Heat-Stable
GBS: Guillain-Barre syndrome
LOS: Lipo-Oligosaccharide
HSP: Heat Shock Proteins
MLST: Multilocus sequence typing
mCCDA: modified charcoal-cefoperazone-deoxycholate agar
ST: Sequence Types
CC: Clonal Complexes
iTOL: Interactive Tree of Life
